# Orphaning stunts growth in wild African elephants

**DOI:** 10.1093/conphys/coac053

**Published:** 2022-07-30

**Authors:** Jenna M Parker, George Wittemyer

**Affiliations:** San Diego Zoo Wildlife Alliance, 15600 San Pasqual Valley Road, Escondido, CA 92027, USA; Save the Elephants, Marula Manor, Marula Lane, Karen, Nairobi 00200, Kenya; Save the Elephants, Marula Manor, Marula Lane, Karen, Nairobi 00200, Kenya; Graduate Degree Program in Ecology, Colorado State University, 102 Johnson Hall, Fort Collins, CO 80523, USA; Department of Fish, Wildlife and Conservation Biology, Colorado State University, 1474 Campus Delivery, Fort Collins, CO 80523, USA

**Keywords:** von Bertalanffy, stunting, orphan, growth, conservation, African elephant

## Abstract

Orphans of several species suffer social and physiological consequences such as receiving more aggression from conspecifics and lower survival. One physiological consequence of orphaning, stunted growth, has been identified in both humans and chimpanzees, but has not been assessed in a non-primate species. Here, we tested whether wild African elephant orphans show evidence of stunted growth. We measured individually known female elephants in the Samburu and Buffalo Springs National Reserves of Kenya, with a rangefinder capable of calculating height, to estimate a von Bertalanffy growth curve for female elephants of the study population. We then compared measurements of known orphans and non-orphans of various ages, using a Bayesian analysis to assess variation around the derived growth curve. We found that orphans are shorter for their age than non-orphans. However, results suggest orphans may partially compensate for stunting through later growth, as orphans who had spent a longer time without their mother had heights more similar to non-orphans. More age mates in an individual’s family were associated with taller height, suggesting social support from peers may contribute to increased growth. Conversely, more adult females in an individual’s family were associated with shorter height, suggesting within-group competition for resources with older individuals may reduce juvenile growth. Finally, we found a counterintuitive result that less rainfall in the first 6 years of life was correlated with taller height, potentially reflecting the unavoidable bias of measuring individuals who were fit enough to survive conditions of low rainfall as young calves. Reduced growth of individuals has been shown to reduce survival and reproduction in other species. As such, stunting in wildlife orphans may negatively affect fitness and represents an indirect effect of ivory poaching on African elephants.

## Teaser

We compared the height of wild female orphan and non-orphan African elephants, finding that orphans are shorter for their age than non-orphans. Stunted growth suggests elephant orphans may suffer reduced fitness. Our results further indicated the presence of peers may increase juvenile elephant growth, while competition with adults may reduce growth.

## Introduction

Post-weaning maternal care is vital to the well-being of many social mammal species, providing benefits to offspring such as defence against predators and conspecifics, provisioning and knowledge of social and ecological landscapes (Clutton-Brock, 1991). The loss of such care results in lower survival for weaned orphans of species such as African elephants (*Loxodonta africana*), spotted hyenas (*Crocuta crocuta*), red deer (*Cervus elaphus*), chimpanzees (*Pan troglodytes*) and orcas (*Orcinus orca*) (Watts *et al.*, 2009; Foster *et al.*, 2012; Andres *et al.*, 2013; Stanton *et al.*, 2020; Parker *et al.*, 2021). In the case of at least one species (the African elephant), this lowered orphan survivorship depresses population growth (Parker *et al.*, 2021), making orphaning and the consequences of being orphaned relevant to conservation.

Weaned orphans who manage to survive may also suffer consequences that could lead to reduced fitness. For example, wild chimpanzee orphans secreted more stress hormones than non-orphans for a period of time following their mothers’ deaths (Girard-Buttoz *et al.*, 2021); African elephant orphans suffered more aggression from conspecifics and had less access to mature adult females who are repositories of social and ecological knowledge (McComb *et al.*, 2001; Wittemyer *et al.*, 2007b; Foley *et al.*, 2008; Goldenberg and Wittemyer, 2017, 2018); and orphans of primate species including muriquis (*Brachyteles hypoxanthus*), blue monkeys (*Cercopithecus mitis*) and savannah baboons (*Papio cynocephalus*) have had lower reproductive success because their offspring were more likely to die than the offspring of non-orphans (Zipple *et al.*, 2021). Evidence also suggests humans (*Homo sapiens*) whose mother has died have stunted growth when compared to humans with both parents, but the degree of stunting was influenced by their social situation, for example if the surviving father lived with a maternal orphan their growth was less stunted (Finlay *et al.*, 2016).

Similar studies of orphan stunting in wildlife are sparse, likely because it is challenging to noninvasively measure growth of known wild individuals. Yet such studies are valuable because stunted growth reliably indicates reduced fitness (Johnson, 2003; Altmann and Alberts, 2005; Dmitriew, 2011; de Onis and Branca, 2016). Animals who can allocate energy to growth tend to be in better condition and have access to more resources (Altmann and Alberts, 2005; Dmitriew, 2011). Larger individuals are also more likely to survive attacks and have more surviving offspring (Johnson, 2003; Dmitriew, 2011). Despite logistical challenges, at least two studies of wild primates have succeeded in relating growth to maternal characteristics. Researchers showed that growth in wild juvenile baboons positively correlates with maternal rank by placing weighing scales in areas frequented by study subjects (Johnson, 2003; Altmann and Alberts, 2005). Another study used urinary creatine, a by-product of metabolism in muscle tissue, to show that non-orphan chimpanzees (*P. troglodytes*) excreted more creatine than orphans, and therefore that prolonged maternal care is important for immature chimpanzee growth (Samuni *et al.*, 2020).

In this study, we investigated whether the loss of prolonged maternal care stunts growth in a non-primate species, the African elephant. The African elephant population that uses the Samburu and Buffalo Springs National Reserves of Kenya has been closely monitored since 1998, such that over 1000 elephants are individually identified (Wittemyer, 2001; Wittemyer *et al.*, 2013, 2021). Increased poaching in the population from the years 2009–2013 left many individuals orphaned, and these orphans have been the focal point of several studies assessing the consequences of orphaning in a highly social species of conservation concern (Goldenberg *et al.*, 2016; Goldenberg and Wittemyer, 2017, 2018; Parker *et al.*, 2020, 2021). Building on these studies, we compared orphan and non-orphan elephant heights, as shoulder height is a known correlate of body mass in elephants (Larramendi, 2015). Male and female African elephants grow at different rates (Shrader *et al.*, 2006); therefore, we included only females in this study. We defined ‘orphan’ as an individual whose mother died while the individual was still immature, with maturity marked by giving birth. Our hypothesis was that the loss of maternal care stunts growth in African elephants as found for humans and chimpanzees (Finlay *et al.*, 2016; Samuni *et al.*, 2020), and therefore predicted orphan elephants would be shorter for their age than non-orphans. We incorporated rainfall in early life and estimated weaning age because available resources and early weaning affect nutrition and growth (Altmann and Alberts, 2005; Dmitriew, 2011; Meale *et al.*, 2015). We also assessed potential effects of an individual’s social environment, including the number of consistently available adult females and age mates, as these are important to juvenile elephant social interactions and physiology (Goldenberg and Wittemyer, 2017, 2018; Parker *et al.*, 2022) and may influence degree of stunting for orphans. Our findings add to the literature on the effects of orphaning for weaned but immature individuals of social wildlife species.

## Methods

### Study system

The Samburu and Buffalo Springs National Reserves are located at 0.3–0.8°N and 37–38°E in Kenya, separated by the Ewaso Ngiro River and together encompassing 220 km^2^. The ecosystem is semi-arid and prone to drought, with rain averaging ~350 mm per year between the two wet seasons: April–May and November–December (Wittemyer, 2001; Wittemyer *et al.*, 2021). A long-term monitoring project of elephants began in the reserves in late 1997. To date, roughly 1000 elephants, some regular residents and others wet season visitors, have been individually identified in a continually updated photo ID file that distinguishes them according to ear tear patterns, tusk configurations and other unique characteristics such as the presence of a crooked tail (Douglas-Hamilton, 1973; Wittemyer *et al.*, 2007b, 2021). Detailed demographic history is available for each identified elephant because a long-term monitoring team drives throughout the reserves daily to record which elephants are present together in aggregations, as well as births, deaths when individuals have been consistently missing from families (about a third of deaths are confirmed through carcass surveys), estrous females, matings, injuries and other noteworthy events (Wittemyer *et al.*, 2005a, 2013, 2021). We have precise age estimates for individuals born during the study because newborn calves are generally sighted within 3 weeks of birth (Wittemyer *et al.*, 2013). Age estimates for individuals who were present at the start of the study are also reliable, confirmed with dental molds to be within 3 years for 75% of individuals and within 5 years for 95% of individuals (Rasmussen *et al.*, 2005).

### Height measurements

We used an Impulse 200LR laser rangefinder with zoom scope 7003824 (Laser Technology, Inc.), equipped with an attached tripod and bubble level, to measure elephant height in centimetres. We (JP and GW) measured elephants opportunistically because they had to be standing on level ground with a straight front leg such that a vehicle could be parked parallel to their body with an unobstructed view. When an elephant was resting in proper position, we parked with the observer on the side nearest the elephant, situated the tripod on the vehicle window ledge, aligned the rangefinder using the bubble level and measured perpendicular distance to the elephant. Next, ensuring that the rangefinder bubble level remained in place, we pivoted the rangefinder to measure the angle down from the perpendicular to where the distal portion of the elephant’s front foot touched the ground, and then the angle up from the perpendicular to the top of the elephant’s shoulder blade. The Impulse rangefinder is equipped with a function that then calculated height based on trigonometry. In this way, we repeatedly took and recorded as many measurements as possible before the elephant shifted position.

For this study, elephants were measured from 1999 to 2005 and 2011 to 2019 (GW) and 2015 to 2019 (JP). We discarded measurements of an individual if there was only one taken within 6 months, and where measurements taken on a single date were highly variable (i.e. <3 measurements with a range >5 cm, <6 measurements with a range >10 cm, <9 measurements with a range >15 cm or measurements with a range >20 cm). Following these quality control steps, the total number of female elephants measured was 222 (GW 158, JP 75 with some overlap). To remove concerns about interobserver variability, we used only GW’s measurements of 158 elephants to calculate the female population’s growth curve (described below), as he measured more elephants across a larger age range (rounded age range, 1–58 years; mean ± SD = 20.24 ± 14.14 years). Of these elephants, 113 were measured on only one date, 39 on two dates separated by at least 6 months (and usually separated by 3 or more years), and 5 on three dates with at least 6 months between consecutive measurement dates. An average of 5.83 (± SD 2.13) measurements were taken from an individual on a single date. We calculated a median height for each individual on each date they were measured, for a total of 206 median heights used to calculate the growth curve.

When addressing our main question we controlled for interobserver variability by using only JP’s measurements, as she focused on measuring orphans and age-matched non-orphans and included only subjects who were born during the study period to ensure precise age estimates. Therefore, we analysed 611 total measurements from 59 elephants of 28 families, consisting of 32 orphans and 27 non-orphans aged 6.87–19.61 years (mean ± SD = 13.85 ± 2.78 years), and compared these data according to the derived growth curve (see below). Some of the 59 elephants were measured on more than one date at different ages, with mean of 2.00 (±1.40) dates of measure per elephant, and an average of 5.14 (± SD 3.31) measurements taken from an individual on a single date. We were able to include all 611 measurements in our primary analysis (described below) regardless of the number of days separating measurement dates for an individual and despite variation in the number of measurements taken per individual on a single date, because the analysis model accounted for variation in age and the estimated precision parameter incorporated variation in number of measurements per individual. (See supplementary material to view height measurement data.)

### Determination of growth curve

We paired the 206 median height measurements of 158 individuals with their estimated or known age on date of measure, rounded ages to the nearest year, then used FSA version 0.9.1 (Ogle *et al.*, 2021), FSAdata version 0.3.8 (Ogle, 2019) and nlstools (Baty *et al.*, 2015) packages in Rstudio version 1.4.1106-5 (R Core Team, 2020; Rstudio Team, 2020) to calculate a standard von Bertalanffy growth curve (von Bertalanffy, 1938; Lee and Moss, 1995; Ogle, 2013) for female elephants of the Samburu population (code for this calculation is available in supplementary materials). The formula for the curve was ${h}_i={H}_{\infty}(1-{e}^{-K(a-c)})$, where ${h}_i$ is the height of an individual, ${H}_{\infty }$ is the estimated average asymptotic height reached by individuals in the population, $K$ is the growth rate coefficient, $a$ is age and $c$ is a constant calculated to achieve the best fit to data under curve constraints (von Bertalanffy, 1938; Ogle, 2013).

### Calculation of covariates

We included six covariates for the 59 individuals in our primary analysis. The first covariate was (i) rainfall. We summed rainfall (mm) recorded the month prior to an individual’s birth and over her first 6 years of life as measured in Archer’s Post, a town on the Samburu National Reserve’s eastern boundary. Rainfall during the first 2–3 years captured conditions during the most dependent period of a calf’s life (Lee and Moss, 1986), and we included as many additional years as possible given the age of the youngest calf measured was 6.87 years old. We included rainfall the month prior to birth to approximate conditions at birth because vegetative growth lags behind rainfall.

The next four covariates captured aspects of juvenile social status: (ii) orphan status (binary variable: 0 non-orphan, 1 orphan); (iii) years spent without mother, calculated by subtracting the mother’s estimated death date from date of measure and dividing by 365 such that partial years were represented by a decimal amount (non-orphans had a value of ‘0’ for this covariate); (iv) the number of adult multiparous females within an individual’s core group (Wittemyer et al., 2005b; Parker *et al.*, 2022); and (v) the number of age mates within an individual’s core group (Parker *et al.*, 2022). To define associate adult females and age mates, we used long-term monitoring data for the 10 years between 2009 and 2019 to calculate association indices between each subject and each member of her extended family. Association index (AI) between two individuals A and B was calculated as follows:

$$ AI=\frac{N_{AB}}{{\mathrm{N}}_{AB}+{\mathrm{N}}_A+{\mathrm{N}}_B}, $$

where N_AB_ is the number of times individual A and B were seen together, N_A_ is the number of times individual A was seen without individual B when B was available in the study population and N_B_ is the number of times individual B was seen without individual A when A was available in the study population (Goldenberg *et al.*, 2016). We only used observations for which observers were confident they recorded all individuals present in a group (Wittemyer *et al.*, 2005b) and only observations taken during the wet seasons of April–May and November–December to ensure seasonal differences in reserve use did not influence interpretation of social units (some measured individuals typically only came to the reserves in wet seasons when elephants group together in larger aggregations). Our multiparous female covariate was defined as the number of females who had given birth at least twice with which a subject had an AI of ≥ 0.5. Our age mate covariate was the number of associates within ±4 years of age of the measured individual with which she had an AI of ≥ 0.5, as 4 years is the average interbirth interval for a Samburu elephant female (Wittemyer *et al.*, 2013).

Finally, we included a covariate of (vi) weaning age. We estimated this covariate by using an individual’s age when their first younger sibling was born. This is an imperfect estimate as some mothers have been observed attempting to wean their calves before a younger sibling was born, and there have been infrequent observations of older calves nursing even after a younger sibling was born. Additionally, for one individual of a family that is not resident to the study area, observers may have missed counting a younger sibling that died shortly after birth. This individual was 14.12 years when her next youngest recorded sibling was born, therefore we capped weaning age at 6.5 years, slightly above the maximum age of 6.35 years for which we are certain we observed the next youngest sibling. We also capped weaning age at 6.5 years for an orphan of another family not resident to the study area whose mother died when she was 9.92 years old without a record of a younger sibling. Finally, in one case a 3.78-year-old individual’s next youngest sibling died less than a month after birth and she may have resumed suckling, but we kept her weaning age at 3.78 years. The weaning estimates ranged from 2.73 to 6.5 years, mean 4.23 (± SD 0.80) years. Only three of the included orphans lost their mother before a younger sibling was born, at ages 3.68, 4.42 and 5.75 years.

### Statistical analysis

We analysed the influence of (i) rainfall in the first 6 years, (ii) orphan status, (iii) time since mother’s death, (iv) number of adult females, (v) number of age mates and (vi) estimated weaning age with a Bayesian hierarchical regression model (Hobbs and Hooten, 2015) fitted on the von Bertalanffy growth curve estimated for the population, using uninformative priors for all parameters (see supplementary materials). The first level of the model organized individuals by family, estimating a family-level asymptote (${\alpha}_{hi}$) to account for genetic constraints on the height that an individual can reach:

$$ {\alpha}_{hi}=240.135+{\alpha}_h, $$

where 240.135 is the H_∞_ parameter determined from the fitted growth curve (see results) and *h* is an index from 1–28 according to the family of individual *i*. The resulting asymptotes (α*_hi_*’s) were incorporated into the second level of the model that estimated height for individual *i* on date *l* according to measurement *j* (μ_ilj_) by incorporating their age (a) and adding the other covariates of b = first 6 years of rainfall, o = orphan status, t = years spent without mother, f = number of multiparous females in core group, m = number of age mates in core group and w = estimated weaning age:

\begin{align*} {\mu}_{il j}&={\upalpha}_{hi}\left(1-{\mathrm{e}}^{-0.198\left({a}_{il}+2.166\right)}\right)+{\beta}_1{b}_i+{\beta}_2{o}_i+{\beta}_3{t}_{il}+{\beta}_4{f}_i\\
&\quad +{\beta}_5{m}_i+{\beta}_6{w}_i, \end{align*}

where 0.198 and −2.166 are the K and c parameters determined from the fitted growth curve (see results). Following examination of a histogram of the height measurement data (y_ilj_) we assigned it a normal distribution:

$$ {y}_{ilj}\sim Normal\left({\mu}_{ilj},\tau \right), $$

where τ is a precision parameter associated with uncertainty surrounding measurement and the process by which height is determined.

We standardized all covariates prior to analysis by subtracting mean and dividing by SD ($\frac{x-\overline{x}}{\upsigma\ }$) and ran our analysis in RStudio version 1.1.463 (R Core Team, 2020; Rstudio Team, 2020) with the package *rjags* version 4-10 (Plummer, 2019) using Markov-Chain Monte Carlo and three parallel chains of 100 000 iterations. We used 1000 iterations for adaptation and discarded 10 000 iterations as burn-in. We examined post-burn-in chains of the model using the *MCMCvis* package (Youngflesh, 2018), calculated Gelman-Rubin diagnostic Rc values (Gelman and Rubin, 1992; Brooks and Gelman, 1998) and ran the Heidelberger–Welch test (Heidelberger and Welch, 1983) to test for convergence. We also assessed model fit by simulating data according to our top model and overlaying a plot of the simulated data and real data with the package *bayesplot* (Gabry and Mahr, 2020) (see supplementary materials for all analysis and model check code). Figures were made with the *ggplot2* (Wickham, 2016) and *MCMCvis* (Youngflesh, 2018) packages.

## Results

### Growth curve

**Figure 1:**
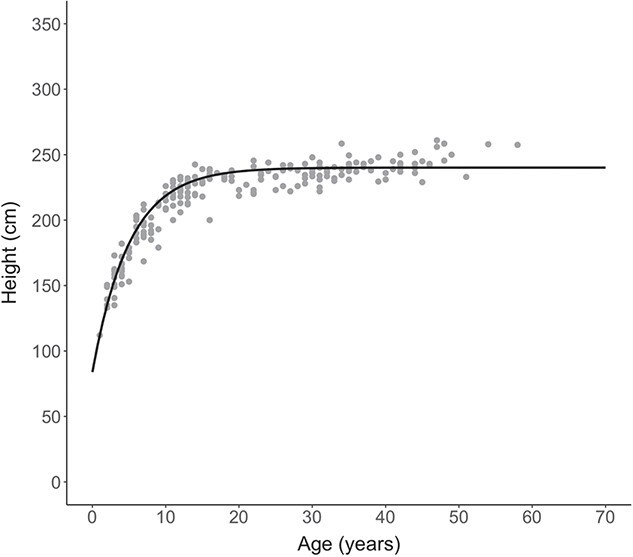
Estimated von Bertalanffy growth curve for the Samburu African elephant female population, with axes scaled to match Lee and Moss (1995) and Shrader *et al.* (2006) for comparison. Grey dots show data points (*n* = 206 median heights from *n* = 158 female elephants aged 1–58 years).

The von Bertalanffy growth curve for female elephants of the Samburu population was

$$ {h}_i=240.135\ast (1-{e}^{-0.198\left(a+2.166\right)}), $$

where ${H}_{\infty }$ = 240.135, $K$ = 0.198 and c = −2.166 (Fig. 1). The curve begins to asymptote between 20 and 30 years, but does not come to a true asymptote until after 50 years (Supplementary Fig. 1) (Lee *et al.*, 2016).

### Primary analysis

Rc values for the analysis model were all <1.1 (Gelman and Rubin, 1992; Brooks and Gelman, 1998), and all parameters passed the Heidelberger–Welch test (Heidelberger and Welch, 1983). There was no concerning lack of fit in the posterior check plot overlaying real and simulated data (Supplementary Fig. S2).

**Figure 2:**
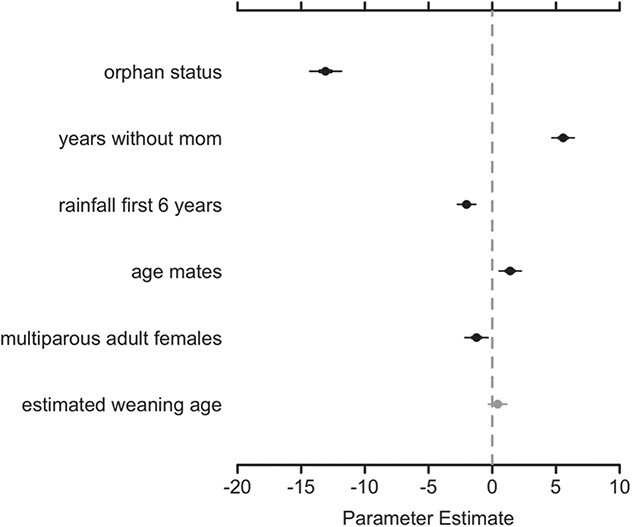
Results from the primary analysis, with 95% confidence intervals, ordered from greatest to least by magnitude of estimated effect. Grey-filled circles signify variables whose 50% confidence interval did not overlap zero, and black-filled circles signify variables whose 95% confidence interval did not overlap 0.

As predicted, orphans were shorter for their age relative to non-orphans, displayed by the negative correlation of the non-orphan/orphan (0/1) covariate with height (Table 1; Fig. 2). Orphans were estimated to be 13.08 cm (with a 95% confidence interval of 11.51–14.35 cm) shorter than non-orphans of the same age, and the orphan status covariate had the largest coefficient value among the covariates assessed. The variable showing the next highest correlation with height was also related to orphaning. Orphans who had spent a longer time without their mother were estimated to be taller for their age than orphans who had spent shorter periods of time without their mother, indicated by the positive correlation of years without mother and height (Table 1; Fig. 2).

**Table 1:** Primary analysis results, ordered from greatest to least by magnitude of estimated effect

Coefficient	Covariate	Estimate	95% CI lower	95% CI upper
β_2_*	Orphan status*	−13.08	−14.35	−11.81
β_3_*	Years without mom*	5.56	4.66	6.45
β_1_*	Rainfall first 6 years*	−2.01	−2.75	−1.28
β_5_*	Age mates in core group*	1.41	0.52	2.29
β_4_*	Multiparous females in core group*	−1.23	−2.16	−0.30
β_6_	Estimated weaning age	0.42	−0.31	1.16

Asterisks denote variables whose 95% confidence interval did not overlap 0.

We unexpectedly found that the amount of rainfall in the first 6 years of life showed a negative correlation with height (Table 1; Fig. 2). The estimated correlation was small, but the 95% confidence interval did not overlap 0. Results also suggest that individuals with more age mates in their group were slightly taller for their age than individuals with fewer age mates, while individuals with more multiparous females in their group were marginally shorter for their age than individuals with fewer multiparous females (Table 1; Fig. 2). The 95% confidence interval for estimated weaning age overlapped 0 (Table 1; Fig. 2).

## Discussion

The benefits of prolonged maternal care are well known (Clutton-Brock, 1991). Studies in wild orphans have underscored its importance by showing that weaned orphans suffer social and physiological consequences, including lower survival (Foster *et al.*, 2012; Andres *et al.*, 2013; Goldenberg and Wittemyer, 2018; Girard-Buttoz *et al.*, 2021; Parker *et al.*, 2021). However, few studies have quantified the costs of orphaning in terms of growth, even though growth is a reliable proxy for fitness and stress (Dmitriew, 2011; Dickens and Romero, 2013). Our results showed orphan elephants were shorter for their age than non-orphans, similar to studies of humans and chimpanzees that showed decreased growth in orphans (Finlay *et al.*, 2016; Samuni *et al.*, 2020).

Our study was correlational and did not determine proximate causes of decreased growth, but at least two factors related to the loss of maternal care were likely involved. First, mother elephants may increase access to food for weaned calves because they reduce displacement by other elephants (Goldenberg and Wittemyer, 2018), thereby increasing access to nutrition and energy available for growth (Dmitriew, 2011). Secondly, increased stress resulting from both the experience of losing a mother and the absence of her care could stunt growth. Reduced body mass and stress are known correlates (Dickens and Romero, 2013; Wu, 2021); the release of glucocorticoids during stress diverts energy away from nonurgent uses like growth (Sapolsky, 2004), and elevated glucocorticoid levels have been associated with lower levels of growth hormones and their binding proteins (Gabbitas *et al.*, 1996; Bernstein, 2010). Previous study suggests orphans do not sustain higher levels of stress hormones ≥2 years after their mother’s death (Parker *et al.*, 2022), but if there are short-term alterations that may affect growth. In chimpanzee orphans, short-term but not long-term alterations in glucocorticoid concentrations were observed (Girard-Buttoz *et al.*, 2021). We note that the majority of orphans in this study were weaned when their mother died (all but three) and we did not find a correlation between estimated weaning age and height, suggesting loss of milk from mothers was not the cause of their stunted growth.

Our results that orphans who had spent more time without their mother were closer in height to non-orphans may indicate that surviving orphans can habituate to the absence of maternal care, likely by strengthening other social bonds (Goldenberg *et al.*, 2016; Goldenberg and Wittemyer, 2017), and have an opportunity for compensatory growth (Dmitriew, 2011). However, compensatory growth can itself be costly (Metcalfe and Monaghan, 2001; Douhard *et al.*, 2017); therefore even if orphans make up for initial stunting following their mother’s death, this does not necessarily equate to an absence of physiological costs associated with reduced growth. Alternatively, older orphans measured in this study generally had survived a longer time without their mother than younger orphans; given the lower overall survival of orphans (Parker *et al.*, 2021), older orphans were likely fitter (and potentially taller) relative to their counterparts that did not survive and therefore were not part of this study.

Rainfall in the first 6 years of life was significantly, but weakly, negatively correlated with elephant growth, a counterintuitive result as early life environmental conditions are recognized as influencing growth in several mammal populations (Plard *et al.*, 2015; Hamel *et al.*, 2016; Veylit *et al.*, 2021). Further, the positive influence of rainfall on growth and fitness has been documented in another African elephant population in Amboseli, Kenya (Moss, 2001; Moss *et al.*, 2011), and rainfall’s effect on elephant demographics such as calf survival has been well established in our semi-arid study system as well (Wittemyer *et al.*, 2007a, 2013, 2021). The drivers of this study’s counterintuitive results are unclear. Possibly, the elephants we measured who were able to survive conditions of drought as young calves represented more fit individuals than others who would have died under similar conditions but were born into more productive times. In addition, natural orphaning disproportionately occurs in low rainfall periods and poaching pressure in the study area increased during a particularly dry period (Wittemyer *et al.*, 2021), causing unbalanced sampling relative to rainfall.

Our findings showed small but interesting correlations between height and the number of multiparous females and age mates within a core group. A previous study found similar relationships between faecal glucocorticoids (stress) and social conditions, notably that social buffering from age mates may reduce stress in wild African elephants (Parker *et al.*, 2022), which could foster growth (Wu, 2021). However, the effect of adult females on the stress response was uncertain (Parker *et al.*, 2022), and our results here suggest the presence of more adult females marginally reduces growth. Possibly adult females increase competition and limit access to resources for growth. Social rank among elephants is strongly correlated with age (Wittemyer and Getz, 2007), meaning adults would outcompete the younger elephants included in our orphan/non-orphan comparison. A greater number of multiparous females also indicates larger group size and thus more individuals to compete with overall. Elephants split into smaller groups during the dry season when there are fewer resources (Wittemyer *et al.*, 2005b), presumably to reduce competition. Our results suggest these fission events may be important for growing juveniles, and thus the need for young elephants to access resources for growth may be a driver of social fissioning in African elephants.

Our study used rich data on elephant individuals from a long-term study, but a notable limitation was our inability to adequately assess genetic effects beyond grouping families together according to a common estimated asymptote. The analysis model could not optimally control for certain families within the study population being generally taller or shorter than others as has been anecdotally observed. We further could not account for paternal effects because paternity is unknown for many individuals in the Samburu population (Rasmussen *et al.*, 2008).

In addition to our findings concerning orphaning, the derived growth curve provides valuable insight into the study population. The Samburu growth curve for females was comparable with that calculated by Lee and Moss (1995) for known-age elephants in Amboseli, Kenya, but indicated a slightly taller population. Lee and Moss (1995) used photogrammetry and a calibrated lens, deriving an H_∞_ of 232 cm when including measurements from animals with estimated older ages and 236 cm when using only animals born during the study period. Our estimate of 240 cm using all data points was similar, and between Amboseli’s asymptotes and the 246 cm asymptote recorded in Kruger National Park of South Africa (Shrader *et al.*, 2006). The work by Shrader *et al.* (2006) demonstrated that elephant growth is similar across Africa; however, other asymptote estimates given in their study were derived from dead elephants on their sides, meaning there was no compression due to body mass and limiting comparability of asymptotic growth across populations other than with the two populations mentioned above.

We recommend similar studies of potential orphan stunting within other long-term projects of terrestrial mammals. Research into possible stunting would be especially interesting in a species in which adoption is common such as the mountain gorilla (Morrison *et al.*, 2021), or with a larger sample size of adopted individuals in chimpanzees as suggested by Samuni et al. (2020). Adopted orphans may grow at the same rate as non-orphans, or at least show less stunting than unadopted orphans. Even more interesting would be if growth correlates with the quality of care provided. In humans, adopted children with a kind caretaker grew at a faster rate than adopted children with a strict caretaker, then growth rates of the two groups flipped when the caretakers switched positions with one another (Sapolsky, 2004).

Stunted growth is relevant to conserving the endangered African elephant (Gobush *et al.*, 2021) because growth positively correlates with fitness (Altmann and Alberts, 2005; Dmitriew, 2011). Poaching, rising human–elephant conflict and increased drought severity due to climate change that remove adult females from elephant populations indirectly decrease population growth by generating more orphans, who have lower survival than non-orphans (Kinyanjui *et al.*, 2020; Gobush *et al.*, 2021; Li *et al.*, 2021; Parker *et al.*, 2021). Our results suggest these factors may have even further-reaching indirect effects by decreasing the fitness of orphans who manage to survive (Dmitriew, 2011).

## Supplementary Material

Web_Material_coac053

## Data Availability

The height measurement data and analysis code used in this manuscript can be found in the supplementary material. Some of the long-term monitoring data are sensitive due to the endangered status of the African elephant, but may be viewable upon request.
